# Autonomic nervous system correlates in movement observation and motor imagery

**DOI:** 10.3389/fnhum.2013.00415

**Published:** 2013-07-30

**Authors:** C. Collet, F. Di Rienzo, N. El Hoyek, A. Guillot

**Affiliations:** ^1^Mental processes and Motor Performance Laboratory, EA 647 CRIS, University of Lyon - Claude Bernard University Lyon 1Villeurbanne Cedex, France; ^2^Institut Universitaire de FranceParis, France

**Keywords:** motor imagery, movement observation, autonomic nervous system activity

## Abstract

The purpose of the current article is to provide a comprehensive overview of the literature offering a better understanding of the autonomic nervous system (ANS) correlates in motor imagery (MI) and movement observation. These are two high brain functions involving sensori-motor coupling, mediated by memory systems. How observing or mentally rehearsing a movement affect ANS activity has not been extensively investigated. The links between cognitive functions and ANS responses are not so obvious. We will first describe the organization of the ANS whose main purposes are controlling vital functions by maintaining the homeostasis of the organism and providing adaptive responses when changes occur either in the external or internal milieu. We will then review how scientific knowledge evolved, thus integrating recent findings related to ANS functioning, and show how these are linked to mental functions. In turn, we will describe how movement observation or MI may elicit physiological responses at the peripheral level of the autonomic effectors, thus eliciting autonomic correlates to cognitive activity. Key features of this paper are to draw a step-by step progression from the understanding of ANS physiology to its relationships with high mental processes such as movement observation or MI. We will further provide evidence that mental processes are co-programmed both at the somatic and autonomic levels of the central nervous system (CNS). We will thus detail how peripheral physiological responses may be analyzed to provide objective evidence that MI is actually performed. The main perspective is thus to consider that, during movement observation and MI, ANS activity is an objective witness of mental processes.

## Introduction

This paper aims to address Autonomic Nervous System (ANS) correlates of motor imagery (MI) and observation. More generally, we will focus on the more potential wide-ranging relationships between ANS activity and mental processes, required to perceive movement or to form a vivid mental representation of this movement. First, readers may question the association of this subpart of the nervous system with one of the highest human cognitive abilities. Indeed, the main well-known role of the ANS is to regulate vital functions of the organism (Appenzeller, 1990), whereas the observation and the mental representation of an action rely on cognitive brain functions (Jeannerod, 1997). The overall activity of the ANS is maintaining the homeostasis of the organism by adapting targeted physiological responses to both the demands of the internal milieu (e.g., postural changes or physical activity) and the changes in the environment (e.g., temperature, altitude, and microgravity).

While observing an action is a bottom-up process leading to perception, we define MI as a centrally controlled movement representation without any associated overt action. Observing a motor scene could thus lead to its mental representation and help the recall of memorized information. MI is mainly based on several sensory modalities, thus associating mentally evoked information (exteroceptive and proprioceptive) to those stored by the procedural long term memory (Decety and Grezes, 1999). According to Kosslyn (1988), two classes of processes are used to form mental images, ones that activate stored memories of the appearances of parts and one that arrange parts into the proper configuration.

Based on this first analysis, the question is “how could we establish a functional relationship between ANS functioning and the ability to observe and to mentally rehearse movements”? In other words, how mental processes, without any associated concomitant movement, could elicit ANS responses? One of the easiest ways to respond is to consider the preparation phase of an action. Anticipated cardio-vascular and respiratory adaptations are well-known physiological processes to face the forthcoming expenditure of energy. At the same time, one should recall the motor plan and adapt the execution parameters to the context in which the movement will be performed, i.e., among others, programming movement force, amplitude and direction (Paillard, 1982). We may hypothesize that, during this phase, we also recall the expected feedback usually provided by actual movement execution, both at the somesthetic (body sensations such as tactile or proprioceptive information) and the environmental levels (the effect of movement upon physical environment). The idea developed by Grush (2004) is that in addition to simply engaging with the body and environment, the brain constructs neural circuits that act as models of the body and environment. If we specifically go back to the issue of performing a voluntary movement, we are in the presence of two seemingly decoupled processes, one quantitative aiming at providing energy for muscles, and one qualitative, designed to adapt parameters of movement execution to its goal (Näätänen, 1973).

Now, let us consider two different contexts within this preparation phase. In the first, the movement outcome is incidental, such as a free throw basketball brand in any end game while the team greatly leads. In the second case, the score is very tight and the success of the shot can allow the player to reverse the result and get his team in the lead. The same action within two different contexts will probably generate two different patterns of ANS responses. Thus, the ANS reacts not only when energy is required to perform an action but also when emotional significance is associated with the action. Therefore, there is a first link between physiological responses from the ANS and mental processes, evidenced through the implicit knowledge of action consequences in case of success or failure. Such consequences may be considered because they can be mentally evoked. From this preliminary observation we can emphasize that each mental construct leading to the building of motor representation should be associated with specific ANS patterns (Decety et al., 1991; Decety, 1996; Guillot and Collet, 2005; Collet et al., 2011).

Based on the classic designs of ANS physiology, we will then describe how the scientific relations between mental processes and the ANS were developed. More precisely, we aim to decipher the reciprocal influence between mental processes, i.e., MI and observation, and ANS activity. In particular, we will describe the ANS responses associated with bottom-up processes (sensory intake operations) and address this issue through the illustration of movement observation. The rejection operation rather corresponds to MI as we could hypothesize the existence of a top-down mechanism, based on the activation of the central representation of movements stored by the procedural memory. In turn, the activation of peripheral effectors during MI also seems to be centrally controlled and we will address the issue of simultaneous programming of somatic and vegetative effectors by common central structures (Collet and Guillot, 2009, 2010). Therefore, we will demonstrate that the ANS correlates of observation and MI offer better understanding of the main features of movement representation. Among others, we will then focus on cardiac and electrodermal activity (EDA) with the aim to detail the main features of these physiological variables, easily recordable with unintrusive procedures. We will progressively examine how mental functions may elicit physiological responses at the peripheral level of the autonomic effectors and illustrate our statements by several examples taken from different fields of human activities, such as usual actions, sporting skills, or motor recovery.

## How the autonomic nervous system functioning is usually described?

In this chapter, we will first review the traditional conceptions related to ANS functioning and describe how the evolution of knowledge has enabled the consideration of ANS activity as a witness of cognitive processes. Early understanding about ANS functioning helps to appreciate why ANS activity was disconnected from other functions than those ensuring the maintenance of vital functions through homeostasis.

It is logical to consider that the control of vital parameters is under automated processes, inaccessible to consciousness, and that physiological variations associated with these vital functions remain unrelated to high mental processes. The ANS is composed of visceral afferent pathways, integration centers at the level of the medulla, the brain stem, the hypothalamus and cerebral cortices. The organization of the ANS describes different efferent pathways subdivided into sympathetic, parasympathetic and enteric branches (Appenzeller, 1990) responsible for regulating internal organs function by nervous signals and neurotransmitters releasing (McCorry, 2007). For example, the medulla has been described as the source for basal vasomotor tone for over 100 years (Hilton, 1981). Efferent pathways target internal organs and, in particular, the cardiac muscle, the smooth muscles, as well as the exocrine and endocrine glands. An important point which will be further developed is that these efferent pathways also innerve the skin directly at the interface between the body and the environment. The ANS is also made of afferent pathways, organized in two sub-systems, the oligosynaptic circuits mediating reflex adaptation responses of the visceral systems, and more complex circuits with projections to nuclei in the brain stem and the brain. Information is collected at the level of internal organs and transmitted to the nervous structures which thus receive feedback information from the periphery. For example, the regulation of the heart and peripheral circulation is under the control of centers in the medulla that receive descending input from higher neural areas in the brain and afferent input from mechanically and chemically sensitive receptors located throughout the body (Mitchell and Victor, 1996). Taken together, this basic view leads to a self-regulating system including centers of commands governing peripheral effectors and feedback information regulating the original command. Functionally, the ANS is made of two main sub-systems, the orthosympathetic branch designed to mobilize energy to face emergency situations (catabolic function), and the parasympathetic branch with the opposite function, i.e., restoring and maintaining the resources of the organism at a level compatible with life functions (anabolic function)^1^. This organization is still valid in light of the advances of modern physiology but remains objectionable for, at least, two main reasons:

(1) From an anatomical viewpoint, and with reference to Xavier Bichat (1802), the ANS remains considered a part of the peripheral nervous system, despite well-identified centers within the spinal cord, the brainstem, the diencephalon and cortical areas (Loewy and Spyer, 1990; Saper, 2002). The two subdivisions made to better understand the organization of the nervous system led Bichat to artificially separate the continuous action of the part controlling *“the organic life”* (visceral) and the intermittent action of the other controlling *“the animal life”* (somatic). In other words, the will and the consciousness leading to voluntary actions in relation with the environment are non-ambiguously separated from self-regulating functions of energy supply, control of energy spending and system maintenance.

(2) From a functional viewpoint, the ANS is designed to maintain constant the internal milieu by mean and opposing reciprocal actions of the sympathetic and the parasympathetic branches (Cannon, 1929). As traditionally described, the sympathetic system has a chain of interconnected ganglia close to the spinal cord, each specifically connecting pre-ganglion to post-ganglion fibers, thus sending information to the target organs. Consequently, the information was believed as being spread across the ganglia chain, resulting in overall activation of the organism thus eliciting the well-known sympathetic tone. Although this concept remains valid and well-accepted (Calaresu and Yardley, 1988), we will later see that this old conception must be qualified. The sympathetic function has thus been described as serving the mobilization of bodily resources, e.g., increasing cardiac and respiratory frequencies facilitating oxygen uptake, routing blood from internal organs to somatic muscles in case of movement preparation and execution, thus providing supplementary energy to both brain and muscles. Conversely, the parasympathetic branch function is mainly anabolic and is likely to decrease demands in energy, e.g., bringing the heart rate to basal level^2^.

Hence, the two subsystems of the ANS have been described as being functionally opposite but with complementary organizations, the action of one branch being reciprocally inhibited by the other, depending on the energy supply for each vital function. ANS functioning was thus summarized as the collaboration of two antagonist subsystems, leading to the image of equilibrium between the two and to the concept of sympatho-vagal balance. Despite powerful clinical application in the field of sudden death prevention (cardiovascular risk stratification) through the heart rate variability (HRV) analysis (Pieper and Hammill, 1995; Malliani and Montano, 2002; Montano et al., 2009), this organization needs update.

## New concepts in neurovegetative physiology

During the last 20 years of the XXth and the early years of the XXIth century, advances in neurovegetative physiology led to question the old conceptions related to ANS functioning. It is not simply organized, i.e., only playing the role of a quantitative system mobilizing energy to serve behavioral output. Two fundamental notions have been more specifically highlighted and discussed independently.

### About the sympathetic tone

The well-accepted notion of sympathetic tone (Calaresu and Yardley, 1988) was early questioned on by the teams of Wallin (1981). They were probably among the first to develop a method enabling the recordings of sympathetic nerve activity targeted to the skeletal muscle vasculature with intra-neural microelectrodes. This technique provided a powerful new tool to study fundamental mechanisms of neuro-circulatory regulation in conscious human participants. During the last three decades, micro-neurographic studies have shed new light on the reflex regulation of skeletal muscle sympathetic nerve activity by arterial baroreceptors, arterial chemoreceptors, and cardiopulmonary baroreceptors (Mitchell and Victor, 1996). Direct recordings of the neural sympathetic activity showed specific responses with little cross talk between ganglia. This finding was later confirmed by Wallin and Fagius (1986) for the experimental article and Wallin and Fagius (1988) for the review article, who found that muscle endings are sensitive to variation in blood pressure while skin endings remain silent. Conversely, the sympathetic endings innervating the skin are very sensitive to mental stress, whereas those innervating muscles are not. A series of experiments by Vissing (1997) further provided evidence in support of a selective central motor command, and demonstrated a highly dissociated pattern of sympathetic activation to skin and skeletal muscle. This set of results is in favor of separated subsystems within the sympathetic organization, made of building blocks controlling specific internal functions (Jänig, 1988). Jänig, McLachlan and Häbler confirmed these first results and synthesized the main data within a series of review papers between 1992 and 2003. Though the sympathetic component of the ANS is widely concerned with the body response to stress, they demonstrated that a range of neuroscientific techniques (including micro-neurography, see Vallbo et al., 2004 for a review) revealed the specialized properties of the functional pathways in the sympathetic system at molecular, cellular and integrative levels (Jänig and McLachlan, 1992a,b; Jänig and Häbler, 1999, 2003). Interestingly, these results confirmed that ANS activity was specifically modulated by mental processes such as mental stress (Vallbo et al., 2004). The emergency function of the ANS was thus incorporated into the general concept of stress or arousal. For example, after stressing the participant with a loud noise, direct measurements of muscle sympathetic nerve activity showed a decrease of bursts. Skin sympathetic activity also increased at the onset of static exercise before any rise in direct measurements of muscle sympathetic nerve activity. Thus, during experimental conditions different from rest (e.g., mental stress and exercise), specific changes in sympathetic activity to selected tissues (e.g., skin) occurred while there was no change at the level of others (e.g., muscle). In addition, inter-individual differences were reported in sympathetic responses to arousal (Halliwill et al., 1997; Donadio et al., 2002) and to mental stress (Carter and Ray, 2009). The hypothesis of selective influence of sympathetic outflows was also shared by Kummer (1992), and more recently by Morrison (2001). Let the concluding sentence to Vallbo et al. (2004):

“The early views of the sympathetic nervous system as a monolithic effector activated globally in situations requiring a rapid and aggressive response to life-threatening danger have been eclipsed by an organizational model featuring an extensive array of functionally specific output channels that can be simultaneously activated or inhibited in combinations that result in the patterns of autonomic activity supporting behavior”.

In a series of original publications from 1995 to 2009, Porges underlined that early conceptualization of the vagal function, focused on an undifferentiated efferent pathways, was assumed to modulate “tone” concurrently to several target organs (Porges, 2009).

### The vagal pathways

Interestingly, Porges reported how the internal organization of the vagal pathways changed across phylogenetic transitions in the vertebrates ANS. Specific changes in vagal pathways regulating the heart occurred along the phylogenetic shift between reptiles and mammals. At the neurological level, heart regulation shifted from the dorsal motor nucleus of the vagus in reptiles to the nucleus ambiguous in mammals, leading to two subsystems within the main parasympathetic branch. The fibers originated from the dorsal motor nucleus of the vagus are unmyelinated (dorsal vagal complex) while those from the nucleus ambiguous are myelinated (ventral vagal complex). Interestingly, the first subsystem is the part of the vegetative vagal function *per se*, thus acting as a behavioral moderator, while the second is mostly linked with more attention processes, emotional reactivity and social communication (Porges, 2007a). Thus, Porges drew the same conclusion to that we reported when describing the organization of sympathetic pathways. The Porges' polyvagal theory links the evolution of the ANS to affective experience, emotional expression, facial gestures, vocal communication, and social behavior. There is thus a clear linkage between mental functions and the ANS (Porges, 1995a).

### Sympathetic and parasympathetic relationships

Finally, Berntson et al. (1991) reported that the relationships between the two ANS subsystems are more complex than the principle of simple reciprocity. They showed that coupled reciprocity is only one of eight possible “modes of autonomic control” that determine heart rate. As reported by Backs (1998), faster heart rate could be elicited by multiple autonomic control modes, e.g., reciprocal coupling, or other modes of autonomic control such as uncoupled sympathetic activation or uncoupled parasympathetic inhibition. We could also assume that autonomic activity may change greatly even when heart rate does not vary across tasks with non-reciprocally coupled control modes such as sympathetic and parasympathetic co-activation or co-inhibition. For example, Grossman et al. (1990) reported that mental arithmetic elicited reciprocally coupled sympathetic activation and parasympathetic inhibition, whereas negative emotion-induction elicited sympathetic and parasympathetic co-activation, however with a larger sympathetic than parasympathetic response. This interesting approach to cardiac physiology and its relationships to mental processes experienced a period of intense production in the 1990's and then gradually dried up. Methods to delineate the respective contribution of each system were progressively oriented toward more integrated and more sophisticated treatments of cardiac activity, specifically HRV through temporal, frequential (power spectrum) or non-linear analysis. HRV is the beat-to-beat variability of heart rate and is under the main control of the parasympathetic system as the sympathetic outflow on the heart is too slow to elicit beat-to-beat changes (Jose and Collison, 1970). HRV is probably the most used index to assess vagal activity. HRV is sensitive to physical exercise and decreases with increased exertion (Yamamoto et al., 1991). Interestingly, it has been shown that HRV was also sensitive to mental load and, more generally, to any kind of stressor (see Porges, 2007b; Thayer et al., 2009, 2012, for extensive reviews). In this context, HRV has been related to the activity of the prefrontal cortex (Lane et al., 2009), a set of neural structures controlling cognitive performance. More precisely, the model of neurovisceral integration (Thayer and Lane, 2000; Thayer et al., 2009) details the pathways regulating the cardiovascular system from the frontal cortex and describes how these networks associating cortical, sub-cortical and limbic structures control cognitive performance and executive functions with HRV as the main dependent variable. For example, the vagal activity was indexed by and associated with the functioning of selective attention under load by Park et al. (2013).

This issue was early hypothesized by Hugdahl (1996) who concluded that Autonomic activity that accompanies attention, orienting and learning has demonstrated that the ANS is not simply a “non-cognitive” and automatic part of brain function thus linking the study of mental processes to non-ambiguous and easily measurable changes in ANS activity.

### Bottom-up and top-down mechanisms

One of the first experimental proofs of such a linkage probably originated from the early studies of the well-known orienting response defined as the simultaneous response of both the somatic and the ANS to specific stimuli. Appropriate sensory receptors record specific properties of the stimulus which then result in orienting the body into the direction where this stimulus originated (bottom-up). Simultaneously to orienting behavior, alertness increased, thus eliciting a sympathetic response, e.g., increased heart rate or EDA. Both somatic and autonomic activities thus attest that the individual perceived the stimulus. Interestingly, Sokolov et al. (1963) associated the orienting response to the representations of the world in memory thus suggesting that the mental evocation of the same information without its physical presence could generate the same physiological responses (top-down). Due to its role in alertness, the ANS is very sensitive to stimulus novelty which is better aimed at eliciting strong ANS responses e.g., increased heart rate and blow pressure (LeDoux, 2000), simultaneously with different bodily reactions (“flight or fight” responses). Information to which the individual has often faced would obviously elicit weaker responses or no response due to habituation (Bradley, 2009). If this information is memorized, it is likely to be rapidly recognized during observation by the perceptual function which directly compares its actual features to those previously stored by memory during past experiences. The next step is to consider that any memorized information could be recalled in the absence of any overt stimulus, i.e., mentally evoked as mental image. Finally, we may hypothesize that any information mentally evoked could elicit the same ANS activity than that elicited under actual conditions, i.e., during the observation of actual information. The two processes nevertheless differ as the first results from a bottom-up process while the second further depends on a top-down process. An intermediate phase would suggest that behavioral and physiological responses could be indirectly elicited by pre-cued information. If the presentation of partial information could lead to the recognition of the full information, thus observing pictures or videos is likely to evoke the equivalent mental material stored in memory thus helping the construction of mental representation. The evocation of the movement by viewing the motor scene would probably elicit the same mental state, and thus the same physiological changes as those usually obtained when actually performing the action (Paccalin and Jeannerod, 2000; Bolliet et al., 2005b).

## Human mental processes and the autonomic nervous system

At this stage, we assume that both external stimuli, e.g., observing somebody performing a motor sequence, and internal stimuli like mentally rehearsing an action could elicit ANS responses. A particular class of movements is that related to the activity of face muscles when feeling an emotion or when observing somebody feeling an emotion. These could be accompanied by motor activity of the whole body depending upon the intensity of the feeling.

### Emotion and the ANS

Ekman et al. (1983) early showed that heart rate increased when professional actors observed faces miming each basic emotion non-ambiguously. Specific increase in skin temperature was also recorded when the actors mimed this emotion simultaneously with the observation of an individual feeling anger. Direct recordings from postganglionic sympathetic axons innervating the skin with intraneural microelectrodes confirmed that skin sympathetic nerve activity increased with the observation of both positive and negative emotional images (Brown et al., 2012). The observer can then mentally imagine somebody else feeling the same emotion as that previously observed (external MI). He or she can also imagine him (her) feeling this emotion (internal MI). By applying this reasoning to human movement, we thus delineate four mental processes likely to elicit simultaneous changes in ANS activity:

1. Observation of somebody actually performing a movement or through a video scene (third-person perspective^3^).
2. Observation of self in the process of performing an action through a video (first-person perspective).
3. Representation of somebody performing a movement with previous stimulus induction (or not) with a video (third-person perspective).
4. Representation of self in the process of performing the same motor sequence, without any external pre-cueing to help MI. In this latter case, the mental image is self-triggered and supposed to be associated with specific ANS responses.

Many experimental findings indicate that the observation or the mental representation of emotional events activate the ANS, probably from the amygdala (LeDoux et al., 1988). The amygdala's lateral nucleus receives and integrates the sensory inputs from sensory systems which relay in the thalamic and cortical areas. The central nucleus provides the interface with motor systems controlling specific fear responses in various modalities, including behavioral, autonomic, and endocrine responses. Thus, observing or imagining emotionally significant objects or scenes has identical bodily effects as actually seeing the same objects or scenes. Lang et al. (1993) reported an increase in skin conductance, as well as in heart and breathing rates, when participants viewed pictures of threatening objects. Interestingly, the same changes occurred when the participants visualized these objects. To add to this finding, Kosslyn et al. (1996) found that mental images of aversive stimuli activated the anterior insula, one of the major cortical sites controlling EDA and feedback from the ANS, while Kreiman et al. (2000) recorded increased activity from single cells of the hippocampus, amygdala, enthorinal cortex and parahippocampal gyrus when participants looked at pictures or formed mental images of these pictures. Therefore, some of the cells responding selectively when participants viewed emotionally significant stimuli were also selectively active when they were asked to imagine the same stimuli. Thus, imagery might engage neural structures also involved in perception (for a more exhaustive review, see Kosslyn et al., 2010). This statement is congruent with clinical data, where parallel deficits in imagery and perception were reported (Farah, 1984; De Vreese, 1991; Young et al., 1994). In turn, these neural structures may affect peripheral effectors of the body itself, e.g., heart rate or EDA modulation.

### Observation, attention and the ANS

Paccalin and Jeannerod (2000) reported consistent changes during two experiments where participants actually watched an actor lifting a weight with increasing loads or a walking and running performance on a treadmill moving at increasing speed. Accordingly, changes in respiration rate of the observer were proportional to the effort made by the actor and followed the actor's running speed, especially during accelerated running. The respiration rate also increased linearly with the treadmill speed. These results provided first evidence of ANS correlates during the observation of a motor sequence. Bolliet et al. (2005b) then compared two experimental conditions including the observation of a video sequence of oneself and that of somebody else performing the same movement. While the observation elicited ANS responses different from those recorded at rest, no difference emerged among ANS responses of actual movement execution, self-videotaped observation and video-taped observation of somebody else performing the same movement. The same issue was recently addressed by Brown et al. (2013) who requested the participants to watch a first-person running video, i.e., viewing the action as if they had a camera on their own head. They observed significant increases in heart rate, respiration rate, skin blood flow and burst amplitude of muscle sympathetic nerve activity by comparison with baseline. They did not, however, compare the first-person to the third-person perspective. It could be hypothesized that watching a first-person could lead to more engaged observation that watching a video from a third perspective and therefore evoke stronger ANS activity. Nevertheless, both studies by Bolliet et al. (2005b) and Brown et al. (2013) bring a first argument showing that perception, observation and action share common mental processes (Jeannerod, 2001). Decety et al. (1991) early evidenced that self-representation of walking was subjected to elicit ANS responses. Heart rate and pulmonary ventilation covaried with the degree of mental effort, during the mental simulation of locomotion. This was further confirmed by Papadelis et al. (2007) who revealed that heart rate and respiratory frequency significantly increased during imagery sessions as compared to rest. As a whole, these data suggest that the cognitive processes activated during movement execution are involved to the same extent during movement observation and MI, whatever the experimental condition. Another interesting issue is that the ANS responses were proportional to the mental effort. It was generally weaker when the observed movement was performed with low intensity compared to high intensity. For example, lifting a load of about 50% of own best mark elicited smaller and shorter ANS responses than when lifting a load of 90% of own best mark.

Observation seems obviously linked to attention since we must orient our sensory systems (in particular the visual system) in the direction of information of particular interest and then focus our attention while inhibiting concurrent activities which may be seen as distractors. Rizzolatti et al. (1987) early linked the orienting of attention with ocular movements programming, i.e., the attention needed to observe a given event is adequately oriented when the oculomotor program for moving gaze toward this event is ready to be executed. Publications focusing on central correlates of motor observation have been a resounding success since the discovery of mirror neurons by Rizzolatti et al. (1996). Many articles have been added to the first data from the years 2000 and more than one hundred review papers have been published since the first review by Rizzolatti et al. (1999). More precisely, the ventrolateral premotor cortex and the anterior part of the intraparietal sulcus are strongly activated during the observation of actions in humans (Manthey et al., 2003). Another interesting issue deals with the role of the ventral premotor cortex responding to the observation of mouth actions in language comprehension and hand movements associated with language. This is due to the fact that Broca's area, mediating language production and comprehension in the dominant cerebral hemisphere overlaps, in part, with the human ventral premotor cortex (Binkofski and Buccino, 2006). Therefore, the observation of actions performed with the hands and the mouth both activate the ventral premotor cortex and Broca's area. Functionally this complex network is probably involved in polymodal action processing. This execution—observation matching system is a part of polymodal action recognition system, associated with language processing, thus facilitating communication among humans through verbal and motor messages (see Fiebach and Schubotz, 2006, for a more extensive review of the functional contribution of the ventral premotor cortex and adjacent Broca's area to perceptual, cognitive and motor processing). This redundancy within the central nervous system (CNS) is likely to favor clear and unambiguous communication among peers of the same species. Thus, this central organization should be paralleled by peripheral activity both at the level of the somatic (facial expressions and general body language) and the ANS (variations of heart and respiratory rates, selective vasoconstriction or vasodilatation).

As previously mentioned, these mental processes are likely to elicit the same ANS activity as during actual behavior. This nevertheless remains a working hypothesis which should be tested by specific experimental paradigms. As the observation of movements was early shown to modulate premotor cortex activation (Rizzolatti et al., 1996; Manthey et al., 2003), we could infer that physiological activity should be recorded at the level of autonomic effectors. We could nevertheless hypothesize that these responses should be related to the significance attributed to the observed actions. In the first experiments on monkeys by Rizzolatti et al. (1996), the observation of usual daily activities (observation a peer grasping food, then bringing it to mouth) triggered the activity of the rostral part of monkey ventral premotor cortex (area F5). Interestingly the authors also observed that F5 area remained silent when the food was handled and grasped with a tool whose function was unknown by the monkey. Therefore, the activity of this area is also linked to the meaning attributed to the observed action. Manthey et al. (2003) further underlined that neural activity within this area was also modulated when observing erroneous and senseless actions. While the purpose of this paper was to distinguish brain activations corresponding to the analysis of movements from those related to objects during the observation of actions, we could nevertheless underline that neural activity varies when the observed action makes sense or not, or when the action is not well goal-directed. Several working hypotheses could be drawn considering ANS responses that we might expect to correlate to movement observation, taking its meaning into account. The observation of a movement making no sense would then elicit a strong ANS response corresponding to general alertness which would nevertheless be highly and quickly sensitive to habituation. On the other hand, the observation of a movement stored in memory would elicit longer ANS responses duration due to the time needed for action recognition and its emotional significance. Autonomic markers of action observation thus need further experimental investigations to be better described and understood.

Contrasting with the proliferation of work about the CNS correlates in movement observation, articles dealing with the ANS correlates have been limited and did not receive much attention from the scientific community. Observation is probably based on a process connecting the observed movement onto an internal model of the same movement that could then make the participant simulating that action (Iacoboni et al., 1999). In turn, a simulated action can elicit perceptual activity which resembles the activity that would have occurred if the action had actually been performed (Hesslow, 2002). According to Nyberg et al. (2000), different perceptual activities can elicit perceptual simulations, including observation due to its ability to emulate mental representation of movement simultaneously with its observation (see also Macuga and Frey, 2012). With reference to these theoretical and empirical contributions, we may conclude that perception, observation and mental representation share many common mental processes mainly based upon sensorial perception and information stored by the memory systems (Jeannerod, 2001).

As far as motor action is considered, MI should elicit the same central and autonomic activities as those recorded when the movement is actually performed. There are many examples of central activations by MI in the scientific literature (for review, see Guillot et al., 2012). Ehrsson et al. (2003) nicely showed how MI of voluntary movements of several body segments activated the corresponding body-part-specific motor representations (see also Michelon et al., 2006; Szameitat et al., 2007a,b). Comparing brain activations of actual and imagined movements leads to better understand the process of forming mental representations (Among others, see Hanakawa et al., 2003, 2008; Lotze and Halsband, 2006; Munzert et al., 2009). Meister et al. (2004) reported that actually and mentally playing music on a silent keyboard yielded similar activation of the fronto-parietal network. The matching of brain activation during actual execution and MI is a reliable mean to evaluate the quality of mental representation (Lebon et al., 2012). These data provide arguments in favor of the functional equivalence between a movement and its mental representation (Jeannerod, 1994; Grezes and Decety, 2001).

## The autonomic nervous system: a witness of movement observation and MI

MI is usually defined as a dynamic mental state during which the representation of a given motor act is internally rehearsed in working memory without any overt motor output (Decety, 1996). MI is born from self-mental activity and anyone can generate a mental image by recalling any motor program stored by procedural memory. Unlike observation, MI originates from an internal model, resulting from mental operations of generating sequential actions without any overt movement^4^ (Wolpert and Flanagan, 2001; Davidson and Wolpert, 2005). Thus, the mental image is self-formed and the individual does not necessarily need any external information to generate the representation of an action, i.e., a kind of pre-cueing which could help its construction. More precisely, MI is a mental construct, “*a class of images of one*'*s own bodily movements which are used to simulate or plan for subsequent action*” (Stevens, 2005). Therefore, defining MI in these terms underlines the close relationship between movement representation and motor preparation and prefigures the links that could be established between MI and ANS activity. As previously described, the CNS prepares the motor command while the ANS provides the metabolic resources necessary for its execution (Mogenson, 1977). Motor skills require being planned and programmed before the actualization of these operations leads to motor commands and actual execution (Paillard, 1982). If this is an obvious function of the CNS, motor preparation also involves the ANS for providing resources in energy that makes movement execution possible.

### A specific index of the sympathetic system: electrodermal activity

Among others, EDA was early believed as being closely related to mental states. EDA is one of the oldest physiological indices, early recorded at the end of the XIX^th^ century. Féré (1888) and Tarchanoff (1890) believed that EDA was likely to provide information about mental states. Measuring skin conductance or skin resistance (one being the reverse of the other) is of particular interest since EDA variations result from the activity of the eccrine sweat glands which are only controlled by sympathetic endings through acetylcholine release (Shields et al., 1987). The innervation of sweat glands is thus an exception to the principle of dual innervations. There is no parasympathetic command to sweat glands, certainly because stopping sweating simply occurs when the sympathetic command stops itself. Interestingly, EDA is a direct witness of sympathetic action through sweat release, mainly at the level of palmar and plantar surfaces. Thus, EDA reflects the general arousal of an organism and changes in arousal in response to emotionally significant stimuli from both the individual him (her)self or from the environment. Increased arousal is correlated with skin conductance^5^ increase or skin resistance decrease and is paralleled by cardiovascular changes, e.g., increases in heart rate and blood pressure, decrease in HRV. All these physiological changes give evidence of energy mobilization for the preparation of movement execution.

**Figure 1:**
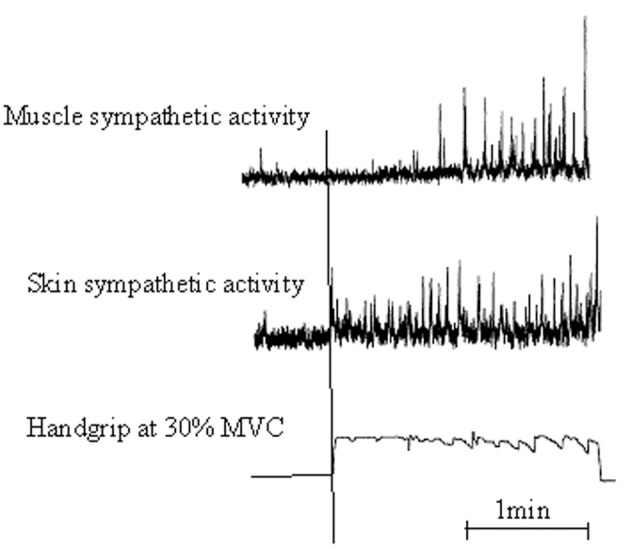
**Illustrative recording from a participant showing a 2-min sequence of static handgrip at 30% maximal voluntary contraction [Adapted with permission from Vissing et al. (1991), Circulation Research]**. Muscle and skin sympathetic nerve activity are recorded from left and right peroneal nerves. Static handgrip markedly increased both skin and muscle sympathetic nerve activity. However, skin ANS increased rapidly and anticipated the onset of handgrip, whereas muscle ANS increased much more slowly with a latency of almost 1 min from the onset of handgrip to the onset of sympathetic activation.

Motor preparation and movement execution are generally associated with EDA increase (Critchley, 2002). These motor-related autonomic responses are mediated, in part, by commands from the CNS, which make the sympathetic arousal varying, according to information significance. As early supposed by Edelberg (1972), results by Vissing et al. (1991), Vissing and Hjortsø (1996), and Vissing (1997) confirmed that sympathetic activation of skin is predominantly influenced by central motor commands (Figure 1). By recording sympathetic nerve activity with microelectrodes placed selectively in skin and muscles during isometric hand contractions, they reported that:

1. Static exercise markedly increases sympathetic outflow to skin as well as to skeletal muscle.
2. The increase in skin sympathetic nerve activity, unlike muscle sympathetic nerve activity, appears to be caused mainly by central command rather than by muscle afferent reflexes.
3. This sympathetic activation of the skin appears to be targeted both to sweat glands and vascular smooth muscle.

At the anatomical level, we thus have several proofs of connection between the CNS and the ANS during the execution of voluntary movements. At the functional level, somatic motor commands are paralleled by a series of autonomic commands targeting skin effectors, i.e., sweat glands and smooth muscles of blood vessels. The resulting effect was to elicit EDA as well as skin blood flow decrease due to skin blood vessels constriction. Thus, EDA may be seen as a sensitive psychophysiological index of changes in autonomic sympathetic arousal that are integrated with sensory-motor, emotional and cognitive states. As previously described, EDA correlates of actual motor command should also be elicited when a motor sequence is simply observed or mentally evoked, i.e., when the participant visualizes the sequences of movements and/or perceives the corresponding body sensations usually. For instance, Oishi et al. (2000) and Oishi and Maeshima (2004) reported a significant decrease in skin resistance associated with increase in heart and respiration rates as early as participants started a MI session, compared to the control condition.

The ANS has features that make this system the natural “first principle” from which initiation of action can arise (Peters, 2000). To give proofs to this assumption, we will first describe how ANS activity is analysed to differentiate a mental state from rest, on the one hand (Falk et al., 2010), and to detect motion execution with a distinction among several movements, on the other (Marchal-Crespo et al., 2013). In the first experiment, Falk et al. (2010) recorded several ANS variables (EDA, skin temperature, heart rate and respiration rate) then processed with hidden Markov models^6^ to automatically detect whether a participant was subjected to perform music imagery as opposed to rest. In the second experiment, Marchal-Crespo et al. (2013) aimed at detecting motion execution by monitoring ANS responses. They first selected a series of four ANS variables which were also processed with the hidden Markov models. The participants were requested to perform several discrete isometric pinching sequences with the aim to detect the pinching periods from rest. Movement execution was accurately classified and separated from rest on the basis of peripheral autonomic signals. Accuracy, sensitivity and specificity reached level of significance. This methodology gives promising evidence for further research on the use of the ANS response in body-machine interfaces.

### ANS recordings and brain-computer interface

When severe and multiple disabilities of the somatic part of the nervous system do not allow individuals to have an access pathway to interface with their environment, controlling the self-regulation of central signals of the ANS may provide a reliable substitute. Peripheral ANS signals can be voluntarily triggered and associated with a brain interface, thus having the potential to be used as an access pathway by the target population (Blain et al., 2008). Body-machine interface may help people suffering from neurological injuries to assist the movement they cannot achieve by themselves. Central signals from MI activity as well as their peripheral correlates, in the form of a set of ANS responses, may be associated to allow participants attempting to trigger robotic assistance. In turn, active assisted exercise provides novel somatosensory stimulation that can help induce brain plasticity (for reviews, see Dobkin, 1993; Rossini and Pauri, 2000; Rossini and Dal Forno, 2004; Dunlop, 2008). Brain-computer interfaces could be designed to control robotic devices which may help to move the impaired limb with the condition that either an intention to move is detected from cortical activity. The physiological change associated with the intention to move should be emphasized if this signal is associated with additional cortical activity generated by MI. Finally, both signals from the CNS system could be linked to peripheral ANS responses these are likely to elicit. Thus, the feasibility of a body-machine interface aimed at detecting motion execution may rely on monitoring the ANS response, an easy and unintrusive method to detect physiological signs from the will. The question of whether specific physiological signs (such as electrodermal responses) are reliable remains open as these can be severely impaired in the event of spinal cord injury, for example. The spinal cord isolated from the brain stem has less or no potential to generate electrodermal responses, as supraspinal connections are necessary for this. More precisely, the integrity of central sympathetic pathways of the upper thoracic segments is required for palmar electrodermal responses and possibly all thoracic segments for plantar electrodermal responses (Yokota et al., 1991; Cariga et al., 2002). If the electrodermal responses are too different due to the variability of spinal cord lesions, the control of a brain-machine interface would be too random. This means that associating several physiological signs together would be necessary to ensure the reliability of a brain-machine interface, this reliability being based on the complementarity and redundancy of the physiological signs. There is an important field of research to explore for years to come^7^.

### ANS recordings and physical practice

Whether ANS responses could be used to evaluate the peripheral correlates of observation or MI has specifically been questioned in the field of sport training and motor rehabilitation. First, the aim was to integrate mental practice to improve motor performance, without any additional physical load.

During the preparation phase of closed skills^8^, the participant is likely to mentally rehearse the motor sequence he (she) will perform within the forthcoming seconds. This period is favorable for studying autonomic correlates of MI. In a study involving elite air-rifle shooters, Deschaumes-Molinaro et al. (1992) showed that the concentration phase before shooting elicited autonomic responses very close to those recorded during the forthcoming actual shooting phase. The almost identical nature of ANS responses during the concentration and the actual shooting periods provided evidence that elite shooters recalled memorized routines of shooting by imagining the forthcoming motor sequences through a top-down process, with the aim to better control the forthcoming execution stages. Interestingly, shooting accuracy was better when ANS activity during the concentration period was close to that recorded during actual shooting (Deschaumes-Molinaro et al., 1991). As a consequence, ANS responses were an important factor of final motor performance and could be used to control mental rehearsal during training sessions.

Tremayne and Barry (2001) recorded electrodermal and cardiac activities during the preparation phase of elite pistol shooters. EDA varied as a function of arousal before shooting, attesting the regulation of energy exertion to specific task requirements. Another interesting result is that the pre-shot electrodermal levels were lower for the best performances, as compared with the worst shots. These variations in EDA were paralleled by a pre-shot cardiac deceleration which was longer and more systematic for best than poor shots. As previously mentioned (Porges, 1995b), cardiac deceleration has clearly been linked with attention processes (for review, see Jennings and Van Der Molen, 2005; Bradley, 2009; Thayer et al., 2009). These critical findings underline that reliable information is obtained from the ANS activity, the first being related to variations of arousal, and the second associated with more qualitative processes such as focusing attention (Näätänen, 1973). Skin conductance tonic level is a good index of arousal level and changes in arousal. Skin conductance response (SCR) corresponds to a rapid time-varying response, i.e., phasic activity, usually recorded in response to various stimuli, either from the environment or directly self-triggered by the participant him (her) self who can evoke mentally a movement. The preparation phase is aimed at adjusting tonic level to be aroused adequately, thus enabling to anticipate and process any information needed to perform well. SCR is elicited by each mental evocation of a movement and its duration is highly correlated to the duration of mental representation.

The fact that MI could elicit tonic changes attesting to variations in arousal may be generalized to any motor sequence. This was evidenced in a usual motor activity like walking. Decety et al. (1991) measured cardiac and respiratory activity during actual and mental locomotion as a function of increasing speeds. Wuyam et al. (1995) also studied locomotion on a treadmill to examine whether MI influenced respiration rate. In both experiments, heart rate and respiratory frequency increased proportionately with the mental effort of the imagery experience. Fusi et al. (2005) also confirmed that imagined walking led to a significant, albeit small (less than 10%), increase in ventilation and oxygen consumption, and to larger increases (up to 40%) in respiratory rate, which was paralleled by a non-significant trend toward a decline of tidal volume. Heart rate and respiratory frequency served at evaluating the mental effort attached to the MI of swimming over a distance of 100 m (Beyer et al., 1990). Both heart rate and respiratory frequency increased during the MI session as compared to the control condition, i.e., rest. As previously stated, ANS activity associated with MI is generally weaker than that observed during preparation to actual execution. Decety et al. (1991) provided evidence that the degree of autonomic activation of a subject mentally running at 12 km/h was comparable to that of a subject actually walking at 5 km/h. This gives some validity to the hypothesis we previously stated that the vegetative activity was attenuated in response to imagined movements by comparison to that recorded during the corresponding actual exercise. Overall, based on theoretical background related to CNS organization and on experiments with ANS data recordings, there is now ample evidence of the close link between mental processes and ANS activity. Bolliet et al. (2005a) showed strong and rapid tonic heart rate variations in a group of elite weightlifters when they were requested to imagine lifting a bar. They recorded similar physiological patterns during imagined as during actual movement, although to a lesser extent by comparison with actual lifting, as previously underlined. Heart rate nevertheless increased by about a mean of 30% as early as weightlifters imagined being called by the referee for lifting. Interestingly, changes in skin conductance paralleled those of heart rate. Skin conductance increased during the same preparation period and was also seen as an index of increased arousal. Autonomic correlates in imagined movements were also reported by Wang and Morgan (1992) in the same type of task (imagining lifting dumbbells), albeit with different autonomic indicators, as respiration rate and systolic blood pressure increased by comparison to the control condition. Finally, two studies (Oishi et al., 2000; Oishi and Maeshima, 2004) drew the same conclusion and underlined larger changes in heart rate and respiration. They also provided evidence of the sensitivity of EDA, although this variable is less-used than those from the cardio-respiratory function. Oishi et al. (2000) elected a 500 m speed skating sprint sequence and compared ANS correlates of MI to those associated with another mental effort, e.g., mental arithmetic, and control (rest). They reported a significant decrease of skin resistance associated with increased heart and respiration rates during both MI and mental arithmetic when compared to rest. Skin resistance respectively decreased during MI and mental arithmetic by about 45 and 40%, with no significant difference between both conditions. Heart rate increased significantly above control values in MI (44.3%) and mental arithmetic (10.3%) with, however, smaller increase during mental arithmetic. They finally observed the same response patterns for respiratory rate. Oishi and Maeshima (2004) compared two performance levels (elite and novice speed skaters) and found larger changes in heart rate and respiration in the elite speed skaters group. They also compared motoneurons excitability (soleus H-reflex) during MI and reported decreased motoneurons excitability in the elite skaters group, in contrast to the non-elite athletes. The authors linked autonomic and somatic changes to the effects of central motor programming, as we previously hypothesized. They finally suggested that the descending neural mechanisms reducing motoneurons excitability were activated when vivid MI was internally performed.

### The coprogrammation of movement by the CNS and the ANS

As a matter of fact, executive functions might result from programming motor commands at both somatic and autonomic levels of the CNS (Thayer et al., 2009), and is therefore co-programmed, as previously suggested by Mogenson (1977). Consequently, MI can be the first stage of movement since preparing the execution can include mental sequences during which several parts of the movement or even the entire motor sequence may be rehearsed. It was thus postulated that MI enabled conscious access to the infra-conscious operations of motor preparation (Jeannerod, 1994, 1995), and positively impacted motor learning (Jackson et al., 2001). The first effect of MI is to make the ANS mobilizing energy as if the movement would actually be performed. Thill et al. (1997) explained the beneficial effects of MI in terms of central programming structures capable of anticipating the metabolic demands of the task. Hence, distinguishing between movement preparation and mental representation of this movement would be difficult, when considering ANS activity. MI is nevertheless believed at eliciting weaker and shorter ANS activity by comparison to movement preparation for actual execution.

To sum, the activation of autonomic effectors during mental simulation of voluntary movements may originate from motor anticipation of energy consumed by the organism (preparation or anticipation of actual exercise, see Thill et al., 1997) and from the central motor operations of planning/programming occurring before the motor command is sent to peripheral effectors through descending motor pathways (action selection and motor adaptation to the environmental context). Interestingly, we may nevertheless underline that the somatic motor commands are inhibited, at least partially during MI (for a review, see Guillot et al., 2012) while those targeting the autonomic effectors are not, although ANS responses are of lower amplitude and shorter duration than those generally observed during the actual movement. There are thus potential structural and functional dissociations among the efferent systems within this co-programming process (for review, see Collet and Guillot, 2009, 2010).

### Outcomes that could be questioned

Several contributions have recently questioned these results. Mulder et al. (2005) recorded EMG activity, heart rate and breathing while participants observed or imagined performing squat leg with a 12.5 kg load in each hand. With the exception of respiratory rate, they found no other evidence of ANS correlates in mental processes since variations were comparable to the control condition during both observing and imaging. In particular, heart rate did not significantly change during movement observation. An important issue is that both experimental conditions, i.e., observation and MI did not elicit cardiac changes. Paccalin and Jeannerod (2000) drew the same conclusion since observing a model performing weightlifting movements did not lead to changes in heart rate whereas breathing increased significantly. As stated by the authors themselves, they had no direct explanation for this dissociation between heart rate and respiration. In contrast, several other studies reported an increase in heart rate during the observation (Brown et al., 2013) or the mental representation of effortful action (Papadelis et al., 2007). Self-imaging running on a treadmill at different speeds made the heart rate increasing, although to a lesser extent than changes in the respiratory function (Decety et al., 1991). While Wuyam et al. (1995) did not evidence any significant change in heart rate compared to rest during the mental simulation of running by trained athletes, respiration rate and total ventilation significantly increased. Interestingly, Mulder et al. (2005) deepened their interpretation and suggested possible arguments explaining why heart rate remained unchanged whereas respiratory rate was. They hypothesized that the global analysis of heart rate activity lacked sensitivity to detect more subtle changes related to MI and observation. With a reference to Althaus et al. (1998), they indicated that HRV would probably be more sensitive in detecting slight changes in heart activity. We recently proposed to integrate several ANS correlates of MI in an index designed to evaluate individual abilities to form mental representations of movements (Collet et al., 2011). Among them, we described how respiratory sinus arrhythmia was likely to represent the mental effort required by movement visualization. Although mental effort might objectively cause increased heart rate, changes in the heart associated with MI might better correspond to a change in the pattern of the cardiac signal, by reducing arrhythmia, for example (Grossman et al., 1990). We know that focused attention reduces the differences between higher and lower values of the cardiac signal, i.e., HRV (Porges, 2007b). This is what might happen during MI without changing basal heart rate. Another working hypothesis could also rely on opposite changes in heart rate due to simultaneous requirements of the intensitive and directional functions. In other words, the mental effort during MI could have a stimulatory effect on heart rate due to sympathetic nervous system activation (intensitive function) and, at the same time, a moderating effect on heart rate through the solitary and ambiguous nuclei of the vagal system (directional function controlling focused attention). As previously described by Backs (1998) this could correspond to a co-activation of both the sympathetic and the vagal systems, leading to unchanged heart rate but to reduced sinus arrhythmia.

A remaining question relates on respiratory frequency and this issue was addressed by Paccalin and Jeannerod (2000) and Mulder et al. (2005). While increase in respiratory frequency may be attributable to action observation and MI of the same action, an alternative hypothesis may also be considered. Changes in breathing may be attributable to non-specific factors rather than the content of the visual effortful scene itself. Mulder et al. (2005) supposed a kind of contamination due to the fact that respiration features were directly observable from the model. In other words, the participants could see and hear the respiration rhythm of the observed model and then match their own respiratory frequency with it. This relies to well-known mimic behaviors occurring automatically in the presence of other individuals, for example, the timing of our walking pace with that of the person walking next to us. According to Rizzolatti and Craighero (2004), observing an action made by another person leads to activity in the motor system of the observer which is comparable to that occurring when the observed action is actually performed. Respiration has several features which may be easily observed and perceived through the visual and auditory systems. Observing a model during effortful action may thus lead our own respiration rate to mirror the rhythm of that observed. Thus, the main issue to be addressed is whether changes in respiration features of the observer could be due to a contamination phenomenon or related to the own characteristics of the visual displaying movements. Results by Paccalin and Jeannerod (2000) brought significance to the second hypothesis. First, respiration rate is sensitive to intake and rejection situations. For example, respiration rate decreases during relaxation, when the individual is focused on his(her) own activity (rejection task and top-down process) whereas it is likely to increase in case of processing information from outside (intake task and bottom-up process). The key argument for associating ANS changes to the observed scene is to demonstrate that respiration is influenced by the intensity of the observed effort. Paccalin and Jeannerod (2000) reported that respiration rate was higher when the participants observed an actor walking at high speed by comparison to low speed. Additionally, they found a linear relationship between respiration rate and the running speed of the actor, when the observed sequence was a constant acceleration from 0 to 10 km/h.

More recently, the existence of potential correlations between mental activity and ANS activity was also questioned by Demougeot et al. (2009). The authors claimed that discrete and effortful imagined movements do not specifically activate the ANS. They took cardiac activity and blood pressure as dependent variables potentially influenced by MI of trunk, legs and wrist movements. Acknowledging that MI of cyclical movements is likely to generate intense autonomic activity, their aim was to test whether the same conclusion could be drawn for discrete and intense movements. The authors reported increased cardiac activity and blood pressure during MI of trunk and legs but not during MI of wrist movement. While actual trunk and leg movements resulted in different physiological reactions due to orthostatic hypotension phenomenon, MI of the same motor sequences elicited similar physiological response. More than 89% of the trials made arterial pressure and heart rate increasing during MI, thus suggesting that ANS activation was a consistent phenomenon, observed in most participants. Due to the orthostatic hypotension, heart rate was significantly greater during trunk than leg movements. Conversely, heart rate increased to a similar extent during MI of both trunk and leg movements. Moreover, actual trunk movements decreased arterial pressure due to central blood volume displacement toward the legs, whereas the reverse phenomenon was found for leg movements, i.e., increase in arterial pressure due to the opposite central blood volume displacement. Demougeot et al. (2009) concluded that if such a specific anticipatory mechanism exists during imagined actions, a differential effect of trunk and leg movements on arterial pressure would be expected, but this was not the case. Combining this result with the fact that no physiological activation occurred during MI of horizontal wrist displacements, the authors concluded to non-specific ANS activity during MI. There are however several shortcomings with such interpretation. Firstly, orthostatic hypotension is caused by gravity during postural changes, especially when going from lying down to standing (Ichinose and Nishiyasu, 2012). When the body position varies, several actions occur involving all parts of the cardio-vascular system as well as the ANS that helps to regulate their function. Orthostatic hypotension is compensated by feedback processes, mainly from the baroreceptors. Peripheral vasoconstriction and increased heart rate are the major cardio-vascular adjustments to orthostatic stress and include part of the reflex response elicited via the carotid sinus, the aortic baroreceptors (arterial baroreflex) and cardiopulmonary stretch receptors (cardio-pulmonary baroreflex). Brainstem ANS centers are thus informed about blood pressure and compensate for its decrease by modulating cardiac activity (increase in heart rate and stroke volume) and peripheral vascular resistance (vasoconstriction) through the sympathetic branch. Such compensatory processes adjust blood pressure from feedback which could not be anticipated by central control. Therefore, it seems obvious that similar response from MI sequence could not occur and parallel those observed during actual trunk movement eliciting orthostatic hypotension. Ichinose et al. (2004) and Kamiya et al. (2005) suggested that the upward resetting of arterial baroreflex control in response to orthostatic stress facilitates the activation of sympathetic nerve activity, thereby contributing to the prevention of postural hypotension. Muscle sympathetic nerve activity progressively increases in response to increasing orthostatic stress through the gradual upward resetting of arterial baroreflex control. Although this mechanism is aimed at preventing orthostatic hypotension, this is not an anticipated process. We must acknowledge that in the particular case of ANS modulations of vital parameters (e.g., maintaining blood pressure within values remaining compatible with vital functions) ANS activity during MI becomes decoupled from that occurring during actual movement. This does not mean that ANS correlates of MI are not specific. A second issue which should be addressed from Demougeot et al. (2009) is related to the absence of ANS activity during MI of wrist movement. Movements involving only a part of a body segment are probably less likely to elicit ANS responses than those requiring whole body actions. Cardio-vascular modulations should probably be of very limited amplitude in this case, thus remaining undetectable. The sensitivity limits of the measuring instruments could also be reached and the response associated with MI could be masked by the general cardiac function, especially if the observation time is very short, such as during flexion-extension of the wrist. This time-window was probably too low, especially for identifying changes in blood pressure, whose variations are of higher inertia than those from EDA. Finally, in the absence of specific purpose, we should also question the aim the participants were able to assign to successive wrist flexion and extension, which are not goal-directed movements. Since ANS responses are strongly associated with the emotional significance of the action, we probably have to gain by focusing on goal-directed movement, with the aim to increase the likelihood of recording specific autonomic responses.

## Autonomic nervous system responses pattern specificity during observation and MI

As we have just seen, one of the main concerns related to ANS correlates of MI probably relies on response specificity. If we hypothesize that this specificity is real, we should observe different ANS patterns depending on whether the participants are engaged in different imagery modalities, such as internal or external MI perspectives. Ruby and Decety (2001) postulated that differences or similarities between self and other representations may be related to the degrees of self-awareness at the neural level (see also Guillot et al., 2009; Lorey et al., 2009). They reported different patterns of central activation depending on whether each participant self-represented the movement as an actor (internal MI) or a spectator (external MI). Both conditions were associated with common activation in the supplementary motor area, precentral gyrus, precuneus and occipito-temporal junction. The contrast between the third-person (spectator) and the first-person (actor) MI revealed activation in left inferior parietal and somatosensory cortices, thus suggesting that these cerebral areas are specifically involved in distinguishing self-produced actions from those generated by others. This question was early addressed to ANS responses during internal and external MI (Wang and Morgan, 1992).

### Motor imagery types and ANS responses

First, results provided evidence of autonomic changes during MI that were identical to those observed during actual exercise. However, respiratory rate, respiratory exchange ratio, heart rate and diastolic blood pressure were similar during internal and external imagery. The structural distinction observed at the central level was not paralleled by specific patterns of autonomic activity. We cannot conclude at ANS response specificity according to MI modality. Brown et al. (2013) recently stated that observing a motor scene from an internal perspective is more likely to generate ANS activity since the participant feels more engaged than when observing the same motor scene from an external perspective, although the authors did not make this comparison. We should nevertheless underline that the variables used are only derived from cardio-respiratory activity and should be better diversified, especially toward the electrodermal variables. This concern was addressed by Di Rienzo et al. (2012) who studied the effect of physical fatigue on the ability to form accurate mental images, both from the external and the internal MI perspectives. By comparing two dependent variables before and after the participants underwent physical fatigue, they reported no effect on external visual imagery while internal visual imagery accuracy was significantly affected. MI time decreased by about 15% as compared with the “without fatigue” condition whereas electrodermal response decreased by about 48%. Interestingly, these changes only occurred for internal visual imagery, during which each participant imagined the motor sequence as an actor, thus associating the somesthetic cues usually perceived during the actual execution to the representation of the motor sequence. Hence, physical fatigue is likely to specifically affect MI accuracy. This might be explained by updating the internal representation of the motor sequence by taking the actual state of the organism into account before engaging in MI. Feedback from muscle state are believed to alter the ability to perform a motor sequence and provide insights to the reciprocal dependence between mental and motor processes, mediated by ANS activity.

### Motor imagery and peripheral metabolic changes

While the general state of the organism may influence the ability to form accurate mental images from the internal perspective, the reverse question could also be asked to test whether mental rehearsal may have the potential to change muscles metabolic parameters. At the level of chemical changes, data do not support the conclusion that MI has changed peripheral metabolic parameters. This issue was early addressed by Decety et al. (1993), who measured muscle metabolism directly using nuclear magnetic resonance spectroscopy during MI sessions. Cardio-respiratory activity was also monitored during both actual and mental leg exercise and increased simultaneously with muscle metabolic changes during actual exercise: drop in phosphocreatine, increase in inorganic phosphate concentrations and fall in intracellular pH to 6.65. End-tidal P_(CO2)_ was unaltered. Under the MI condition, cardio-respiratory activity was comparable to that elicited during actual exercise. Conversely, the metabolic parameters remained unchanged. The end-tidal P_(CO2)_ decreased progressively to about 18% of the resting value during MI due to a greater elimination of CO_2_ during hyperventilation without increase in CO_2_ production. Under comparable experimental conditions, Wuyam et al. (1995) also reported a reduction in end-tidal P_(CO2)_. This result was later confirmed by Fusi et al. (2005). The monitoring of autonomic changes thus demonstrated that cardio-respiratory activation during MI was greater than that required by the increase in metabolic demands. These results are also in favor of dissociation between somatic and autonomic commands, and provide further evidence for structural and functional similarities between MI and actual motor preparation.

To sum, with the exception of studies by Wang and Morgan (1992), Decety et al. (1993) and Di Rienzo et al. (2012), no other experiment has yet been conducted to address the question of ANS response specificity during MI and new experimental designs, involving a pool of ANS variables including cardio-respiratory as well as EDA, should probably challenge this interesting issue. In the two last paragraphs, we described the potential intra-subject differences through ANS activity. Another way to test ANS responses specificity is to compare central and peripheral response patterns through inter-subjects studies, e.g., when MI is performed by participants with high vs. low imagery abilities.

### Inter-individual differences in MI imagery abilities

**Figure 2:**
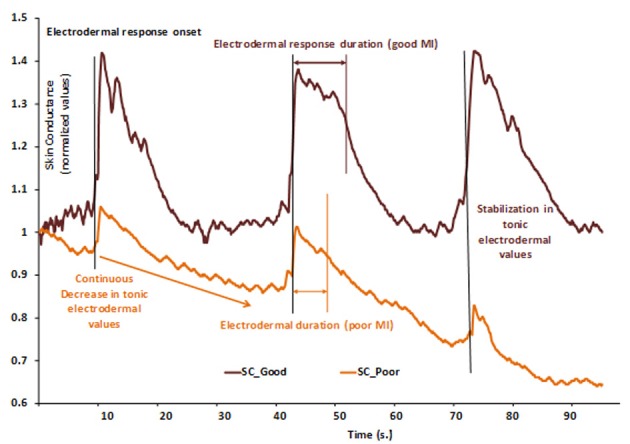
**Example of two normalized skin conductance responses during a series of three consecutive MI repetitions of a finger sequence in skilled (SC_Good, dark brown) and unskilled imagers (SC_Poor, light brown)**. Each vertical bar represents the starting of MI and is paralleled by electrodermal response onset. In the skilled group, electrodermal response duration is comparable to that of the actual finger sequence, thus indicating a close relationship between MI and actual movement durations. Electrodermal tonic values remained at a constant level (around 1), thus attesting that general arousal remained stable and favored general attention. In the unskilled group, electrodermal response is shorter than that of actual movement, thus indicating temporal discrepancy between actual and imagined movement durations. Normalized electrodermal tonic values decreased (from 1.00 to about 0.65), indicating a decrease of general arousal, likely to impair sustained attention. These data suggest that the participant encountered difficulty at keeping arousal at a level that is compatible with efficient cognitive processing. Skin conductance could thus distinguish between MI abilities.

Differences in MI abilities are partially supported by different neural networks (Guillot et al., 2008). The authors compared cerebral activations of skilled imagers to that of unskilled imagers during physical execution and MI. MI abilities were first assessed to assign participants into one of the two groups of skilled and unskilled imagers, using a set of usual tools (questionnaire, mental chronometry and ANS activity recordings). Each group presented a specific pattern of EDA, as illustrated by Figure 2. Both groups were then scanned during the mental rehearsal of a sequence of finger movements and activated a set of common cerebral structures including the inferior and superior parietal lobules and motor-related regions such as the lateral and medial premotor cortex, the cerebellum and the putamen. Interestingly, inter-group comparisons showed differences due to MI abilities: good imagers activated more the parietal and the ventrolateral premotor regions, known as playing a critical role in the generation of mental images. By comparison, poor imagers activated more the cerebellum, the orbito-frontal and the posterior cingulate cortices. ANS responses differentiated MI abilities^9^ and were paralleled by specific central activation, thus attesting specific inter-subjects differences. Interestingly, this result also gives evidence of a close relationships between MI vividness and electrodermal response duration.

## Conclusion

Until the two past decades, the ANS was rather considered to control general physiological changes related to variations in arousal, with no actual link with cognitive operations. If this basic function still remains to serve behaviors related to survival, the modern view of its anatomical organization has enlarged its function to more complex social regulation: “The evolution of the ANS provides an organizing principle to interpret the adaptive significance of mammalian affective processes including courting, sexual arousal, and the establishment of enduring social bonds” (Porges, 1998). Each mental operation including MI is thus reflected within a part of our nervous system which is inaccessible to our will and consciousness, but reveals a part of them in the form of specific and structured physiological variations.

Movement observation and MI are both means to interact with our own environment and represent potential operations favoring the mental construction of an action plan. MI is one of the more sophisticated mental operations making the individuals engaging in predictive activities, drawing plans and anticipating the possible consequences of action planning (Jeannerod, 2001). To react appropriately in social relationships, we also have a tendency to simulate how others think of us through MI. These brain operations are accompanied by a set of physiological information which may obviously be recorded at the central level but also at the level of peripheral effectors. Researchers may thus indirectly evaluate mental states and use an inferential model of brain functioning. Variables from the ANS have a good reliability since these are correlated with mental functioning in a specific way. This association is based on a centrally controlled process activating the central representation of movements simultaneously with ANS regulation during MI (Decety et al., 1993; Fusi et al., 2005). Far from the old and outdated views related to ANS functioning, ANS activity provides evidence of a close correlation with cognition (Hugdahl, 1996; Thayer et al., 2009). The sympathetic outflow to the heart is modulated by the activity of the anterior cingulate cortex, and the cardiovagal activity is under the control of the ventral medial prefrontal cortex (Wong et al., 2007). These two cortical structures are known to control both emotional states and cognition. To the same extent, higher control of EDA is mediated by neural networks involving prefrontal, insular, parietal cortices, and limbic structures including cingulate and medial temporal lobe with the amygdala and the hippocampus (Critchley, 2005). The neural substrate for these peripheral autonomic responses is associated with motivational and affective states which, in turn, mediate action observation and MI. Taken as a whole, recording autonomic variables at the peripheral level provides an open window on high brain functions (Collet and Guillot, 2009). These variables can obviously contribute to the study of MI among other neurophysiological and psychological methods (Guillot and Collet, 2005).

While we underlined the common activations of both central and autonomic nervous systems during observation and MI, these are nevertheless associated with no observable behavior. ANS response amplitude and duration are usually reduced when movements are only observed or mentally performed by comparison with actual execution. There are thus potential structural and functional dissociations among the efferent systems within this co-programming process (for review, see Collet and Guillot, 2009, 2010). Dissociated somatic and autonomic co-programming during MI is a working hypothesis waiting for further experimental investigations. As early hypothesized by Damasio et al. (1996), there is high probability that ANS responses accompanying MI may serve as somatic markers constituting a set of information available to the afferent systems as internal feedback. Finally, with reference to biofeedback theories (Schwartz, 1976), learning to self-regulate ANS response patterns may serve subjective experience and enhance the effectiveness of biofeedback procedures by training the individuals to integrate and coordinate central cognitive information to peripheral autonomic and motor responses. Thayer and Lane (2000) presented a theoretical model integrating central and autonomic networks controlling cognitive and affective functions into a structural and functional system designed to serve self-regulation and adaptability of the organism. This system, including the ventromedial prefrontal cortex and the amygdala, is closely linked with autonomic centers in the brainstem and sustains that HRV may serve as a peripheral index of the integrity of CNS networks that support goal directed behavior. A clear relationship is proposed between autonomic activity (e.g., HRV) and executive functions, this being expected to favor a better understanding of the complex interactions between cognitive, affective, behavioral and physiological factors associated with health and disease. As sophisticated mental processes, observation and mental representation could take place within this model. It is a challenge for the future and an open door to new experiments.

### Conflict of interest statement

The authors declare that the research was conducted in the absence of any commercial or financial relationships that could be construed as a potential conflict of interest.

## Footnotes

^1^The enteric nervous system whose we previously referred to, consists of a mesh-like system of neurons that governs the function of the gastrointestinal system. It will not be described further here because the variables indicating its activity cannot be measured by non-intrusive procedures.

^2^The organization of the ANS is well described in textbooks of general physiology. For a more detailed description, see Appenzeller (1990) “ The autonomic Nervous System ”, pages 3 to 7, Figures 1–6 of Chapter 1 (Anatomy and histology). See also Thayer et al. (2009) for an detailed description of the relationships between central autonomic nervous system centers within the brain.

^3^We previously referred to internal and external MI. It is also usual to distinguish between two MI perspectives, i.e., the spatial perspective from which MI is performed. The first-person perspective is the representation as an actor during which we imagine ourselves performing the movement. The third-person perspective is the representation as a spectator during which we imagine someone else or ourself performing the movement.

^4^A clinical case study showed that brain injury has removed the dissociation between actual performance and mental representation, the patient being no longer capable to inhibit the execution of the movement he was imagining (Schwoebel et al., 2002).

^5^Without specific information, we refer to skin conductance as an index of electrodermal activity.

^6^Hidden Markov Models are used for temporal pattern recognition due to their ability to classify time-sequential data, such as the time-varying physiological signals.

^7^See the publication by Vogt et al. (this special issue), highlighting the growing number of publications on the control of brain-machine interfaces by mental processes such as motor imagery.

^8^Closed skills occur in a stable environment without uncertainty and confrontation with the opponent is indirectly made. Open skills present reverse features.

^9^Sympathetic skin responses were recently used as a quantitative evaluation of motor imagery abilities in individuals with spinal cord injury (Grangeon et al., 2012).
